# Going back to home to die: does it make a difference to patient survival?

**DOI:** 10.1186/s12904-015-0003-5

**Published:** 2015-03-19

**Authors:** Nozomu Murakami, Kouichi Tanabe, Tatsuya Morita, Shinichi Kadoya, Masanari Shimada, Kaname Ishiguro, Naoki Endo, Koichiro Sawada, Yasunaga Fujikawa, Rumi Takashima, Yoko Amemiya, Hiroyuki Iida, Shiro Koseki, Hatsuna Yasuda, Tatsuhiko Kashii

**Affiliations:** Home Palliative Care Committee, Takaoka Medical Service Region, 387-1 Futatsuka, Takaoka, Toyama 933-0816 Japan; Department of Medical Oncology, Toyama University Hospital, 2630 Sugitani, Toyama, Toyama 930-0194 Japan; Department of Palliative and Supportive Care, Seirei Mikatahara Hospital, 3453 Mikataharacho, Kita-ku, Hamamatsu, Shizuoka 433-8105 Japan; Board of Palliative Care, Saiseikai Takaoka Hospital, Toyama Prefecture, 387-1 Futatsuka, Takaoka, Toyama 933-0816 Japan

**Keywords:** Survival time, Home palliative care, Place of care, Palliative care team, Propensity score

## Abstract

**Background:**

Many patients wish to stay at home during the terminal stage of cancer. However, there is concern that medical care provided at home may negatively affect survival. This study therefore explored whether the survival duration differed between cancer patients who received inpatient care and those who received home care.

**Methods:**

We retrospectively investigated the place of care/death and survival duration of 190 cancer patients after their referral to a palliative care consultation team in a Japanese general hospital between 2007 and 2012. The patients were classified into a hospital care group consisting of those who received palliative care in the hospital until death, and a home care group including patients who received palliative care at home from doctors in collaboration with the palliative care consultation team. Details of the place of care, survival duration, and patient characteristics (primary site, gender, age, history of chemotherapy, and performance status) were obtained from electronic medical records, and analyzed after propensity score matching in the place of care.

**Results:**

Median survival adjusted for propensity score was significantly longer in the home care group (67.0 days, n = 69) than in the hospital care group (33.0 days, n = 69; *P* = 0.0013). Cox’s proportional hazard analysis revealed that the place of care was a significant factor for survival following adjustment for covariates including performance status.

**Conclusions:**

This study suggests that the general concern that home care shortens the survival duration of patients is not based on evidence. A cohort study including more known prognostic factors is necessary to confirm the results.

## Background

For cancer patients, dying in a preferred place is one of the most important determinants of quality of life (QOL) [1-4]. Multiple population-based surveys have indicated that approximately half of Japanese people would desire home care during terminal stage cancer [4,5], and a nationwide bereaved family survey revealed that among all patients dying of cancer, around 31% wanted to die at home [1,6-8]. However, the actual figure of cancer patient home deaths during the last decade in Japan was less than 10% [1,7,9]. Thus, the wishes of many Japanese cancer patients do not appear to be met.

The major difference between hospital care and home-based palliative care is the involvement of palliative care specialists. Training for these specialists has recently progressed and is offered mainly by the hospital. However, doctors in the home care are less exposed to these training programs so often have little experience in palliative care, yet have to make important decisions including the best methods for total pain relief, total or peripheral parenteral nutrition, and the permission or prohibition of oral feeding and hydration.

Empirical studies from Western countries have confirmed that when patients die in hospitals or intensive care units, their QOL is often lower than that of patients who die at home, which can increase the risk of psychiatric disease development in bereaved families [2]. By contrast, the shorter admission time to acute hospitals during terminal stage cancer and the greater use of hospice services at home may improve the patients QOL, thus minimizing family mental stress [2].

The Japanese healthcare scheme has not established a general practitioner system like that seen in Europe, and patients have free access to all medical services covered by universal national insurance [1,10]. A typical cancer patient would therefore consult a cancer hospital, university hospital, or large hospital directly, even when a clinic is present nearby. Many Japanese people also believe that hospitals provide a higher quality of care than clinics; for example, parenteral hydration is a minimum requirement of hospitals even in patients whose death is imminent [11]. Patients and their families have often expressed concerns regarding the quality of home care, and many believe that it results in a potentially shorter survival during the terminal stage of cancer compared with hospital care [12].

This study aimed to explore whether the survival duration differed between terminally ill cancer patients who received inpatient care and those who received home care.

## Methods

This retrospective study was based on propensity score matching in the place of care of consecutive cancer patients referred to a palliative care team in a Japanese 270-bed designated cancer hospital (Saiseikai Takaoka Hospital, Toyama, Japan). This study was approved by the Institutional Review Board of Saiseikai Takaoka Hospital. All subjects were adults, and provided their informed consent for study participation.

### Subjects

Subjects included 190 cancer patients who were consecutively referred to a palliative care team between October 2007 and September 2012. We continuously followed the patients from the start of palliative care team intervention until their death or until the end of December 2012, whichever was sooner.

Patients were classified into two groups based on the place of care: (1) the hospital care group (patients who received inpatient hospital care from their referral to the palliative care team until their deaths, without discharge to home), and (2) the home care group (patients who received palliative home care for at least 1 day from home doctors in collaboration with palliative care teams).

### Interventions

In the hospital care group, the primary responsible physicians were attending hospital physicians other than palliative care specialties such as oncologists, surgeons, and physicians certified in medical subspecialties. The hospital palliative care consultation team provided regular daily monitoring, and was available 7 days a week and 24 hours a day on demand.

In the home care group, the primary responsible physicians were home physicians in the community. The hospital palliative care consultation team provided regular weekly monitoring, and was available 7 days a week and 24 hours a day on demand. Additionally, information was shared through one pre-discharge multidisciplinary conference and via structured data sheets (information-sharing instruments) [13]. Visiting physicians, nurses, pharmacists, medical social workers, and other medical staff completed the data sheets with comments for patients and relatives at each visit.

In both groups, palliative care was provided by a multidisciplinary care team, including physicians, nurses, pharmacists, medical social workers, dietitians, and physical therapists. The major differences between the groups were therefore the primary physician and frequency of regular palliative care consultation.

### Measurements

We retrospectively investigated the place of care and duration of survival after referral of the patient to the palliative care team using data from electronic medical records. Additionally, as covariates, we investigated the primary site, gender, age, history of chemotherapy from 1 month before referral to the time of death, and the Eastern Cooperative Oncology Group (ECOG) performance status at the time of referral [14]. Palliative care physicians had prospectively recorded patients’ performance statuses at the time of referral to a palliative care team as routine practice. We counted the number of completed records (as an alternative indicator of the number of medical staff visits) to estimate the intensity of home care only when relatives allowed us to use the structured data sheets after the patient’s death. The palliative care team intervention duration matched the patient survival time after intervention because we continuously followed the patients until their deaths.

### Propensity score model

The propensity score was estimated using a logistic regression model adjusted for primary site, gender, age, history of chemotherapy, and ECOG performance status. These variables were previously shown to be prognostically significant [15]. The matching algorithm on the propensity score was nearest neighbor matching with a ±0.04 caliper and without replacement. We used the standardized difference to measure the variable balance, whereby a standardized difference above 0.1 represented a meaningful imbalance. Standardized differences (d) were calculated using the following formulae:

<In the case of scale data>

$$ \mathrm{d}=\frac{\left|\overline{X_t}-\overline{X_c}\right|}{\sqrt{\frac{{S_t}^2+{S_c}^2}{2}}} $$

${\overline{X}}_t$: the mean of the home care group, *S*_*t*_: the standard deviation of the home care group,

${\overline{X}}_c$: the mean of the hospital care group, *S*_*c*_: the standard deviation of the hospital care group

<In the case of binary data>

$$ \mathrm{d}=\frac{\left|{\widehat{P}}_t-{\widehat{P}}_c\right|}{\sqrt{\frac{{\widehat{P}}_t\left(1-{\widehat{P}}_t\right)+{\widehat{P}}_c\left(1-{\widehat{P}}_c\right)}{2}}} $$

${\widehat{P}}_t$: the probability in the home care group, ${\widehat{P}}_c$: the probability in the hospital care group

### Statistical analyses

The survival duration of patients in both groups was compared using the log-rank test. Survival was defined as the time from first referral to the palliative care team until death. To adjust for factors that might have influenced prognosis, we conducted a multivariate analysis using the Cox proportional hazard model, in which patient background or propensity score were included as covariates. Univariate comparisons were performed using the Student’s *t*-test, Mann–Whitney *U*-test, or chi-square test, as appropriate. The significance level was established as 0.05. All statistical analyses were conducted using IBM SPSS® Statistics version 22 software (IBM Japan, Ltd. Tokyo, Japan.).

## Results

A total of 94 patients were included in the home care group and 96 in the hospital care group; four and one patient from each respective group were still alive during the observation periods. The median follow-up time by the palliative care team was 57.0 days (range, 35–113 days) in the home care group and 33.5 days (range, 16–67 days) in the hospital care group. After propensity score matching, we analyzed 69 patients in both groups. Characteristics of all patients and propensity-matched patients are summarized in Tables 1 and 2, respectively. No significant differences were observed between the background factors of all patients, and no meaningful imbalance was observed following propensity score matching.

**Table 1:** **Characteristics of all patients**

**Item**	**Home care group (n = 94)**	**Hospital care group (n = 96)**	**P**	**Standardized difference**
Gender (n, male/female)	46/48	55/41	0.25	0.17
Age (yr, mean ± SD)	72.7 ± 13.9	69.3 ± 10.9	0.06	0.28
Primary site (n)			0.45	
Lung	12	19		0.19
Stomach/esophagus	31	30		0.04
Liver/biliary tract/pancreas	25	23		0.06
Large intestine	16	9		0.23
Breast	2	4		0.12
Others	8	11		0.10
Performance status (n)			0.20	
0	0	6		0.37
1	13	23		0.26
2	34	23		0.27
3	39	32		0.17
4	8	12		0.13
Use anti-cancer agents (n, used/unused)^a^	3/91	3/93	1.00	0.004

^a^This is about whether patients used anti-cancer agents or not from 1 month before referral to Palliative Care Team to death.

**Table 2:** **Characteristics of propensity-matched patients**

**Item**	**Home care group (n = 69)**	**Hospital care group (n = 69)**	**P**	**Standardized difference**
Gender (n, male/female)	35/34	36/33	0.87	0.03
Age (yr, mean ± SD)	70.7 ± 13.9	70.7 ± 10.8	0.99	0.002
Primary site (n)			1.00	
Lung	11	13		0.08
Stomach/esophagus	22	23		0.03
Liver/biliary tract/pancreas	17	15		0.07
Large intestine	9	8		0.04
Breast	2	2		0
Others	8	8		0
Performance status (n)			0.99	
0	0	0		(incalculable)
1	12	12		0
2	22	20		0.06
3	27	28		0.03
4	8	9		0.04
Use anti-cancer agents (n, used/unused)^a^	1/68	2/67	1.00	0.10

^a^This is about whether patients used anti-cancer agents or not from 1 month before referral to Palliative Care Team to death.

A total of 44/94 and 17/69 structured data sheets were available for all home care patients and the matched cohort, respectively, and the average number of records completed during home care (mean ± SD) was 41.3 ± 35.8 in all patients and 34.1 ± 25.0 in matched patients.

**Figure 1:**
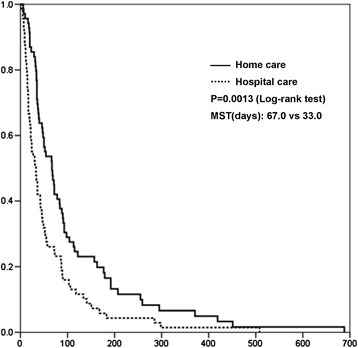
**Kaplan–Meier curves for adjusted overall survival duration of propensity-matched patients stratified according to the settings of palliative care.** The median overall survival times were 33.0 and 67.0 days for patients in hospital and home care, respectively.

The log-rank test showed that matched patient survival time was significantly longer in the home care group (home care group: median, 67.0 days, range, 35–115 days vs. hospital care group: median, 33.0 days, range, 15–72 days, *P* = 0.0013; Figure 1). Cox’s proportional hazard analysis revealed that the place of care was a significant factor in predicting patient survival in all models (Table 3).

**Table 3:** **Cox proportional hazard analysis of home care and mortality**

**Model**	**Hazard ratio**	**95% CI**	**P**
All patients			
Unadjusted	0.61	0.46 – 0.82	<0.001
Adjusted for age, gender, and performance status	0.53	0.39 – 0.72	<0.001
Propensity-matched patients			
Unadjusted	0.58	0.41 – 0.81	0.002
Adjusted for propensity score	0.57	0.41 – 0.80	0.001

CI: confidence interval.

## Discussion

The most important finding of this study was that survival in the home care group was significantly longer than in the hospital care group after adjustment for other factors, such as performance status.

Several studies have previously examined the relationship between place of death and survival in terminally ill cancer patients [2,16,17]. In general, patients receiving home care have been shown to survive for longer than those who received hospital care [17]. However, a recent survey reported no significant difference in survival between the two categories of patients [2]. A systematic review of palliative home care showed that the prognosis of patients receiving palliative home care was more favorable in some studies, whereas no difference was observed in others [18]. Moreover, several randomized controlled trials compared survival between home and hospital care [19-21], but none of them showed a significant difference between care types.

This study adds to the current body of knowledge on the potential relationship between survival and place of care, and confirms that most patients receiving home care had significantly favorable, or at least no worse, survival profiles than those receiving hospital care. The strengths of this study include the investigation of survival time as a primary end-point, and the adjustment for factors influencing prognosis (such as performance status). This study is also the first about this topic in Asian patients who typically prefer hospital rather than home care because of the concern that the latter could shorten their survival [11,12]. Our findings that the home care group had a significantly longer survival than the hospital care group could help to dispel unsubstantiated concerns about the negative effects of home care on patient survival.

One of the potential interpretations of longer patient survival at home is that the home care group had better health than the hospital group. Although we adjusted for performance status, which is one of the strongest prognostic factors, this study was retrospective and investigated only a limited number of prognostic variables. Dyspnea, cachexia-related symptoms, and other independent prognostic factors were not measured, and it is conceivable that we failed to demonstrate the potential backgrounds of patients in the two different treatment settings. Another interpretation is that staying at home might itself minimize patient distress through increasing QOL, resulting in an improved immune system [22], which could lead to improved survival. Alternatively, the patients who received home care may have had a better understanding of their disease status through facilitating patient comprehension of the disease trajectory, which could have minimized potentially harmful medical interventions close to their death [22]. These interpretations should be tested in a future prospective study.

This study has a number of limitations. First, we could not adjust for prognostic factors other than performance status, such as nutrition, dyspnea, and delirium. Future studies should compare survival intervals after adjusting for proven prognostic factors using the Palliative Prognostic Index [23] or Palliative Prognostic Score [24]. Until then, the survival difference between the two patient groups cannot be confirmed. Second, this was a retrospective survey, so the details of the quality of palliative care are unknown. The data include both patients with advanced cancer at their initial visit and those whose cancer progressed to an advanced state during treatment. Therefore, we cannot precisely calculate the time period from advanced cancer until death. Third, this was a single institutional study with a relatively small sample size, and generalizability may be limited because of potential differences in the availability of community and hospital health care resources. Fourth, neither the timing of the discontinuation of anti-cancer drug regimens nor all of the details of cancer interventions were available [15,25,26]. The results of this study are therefore preliminary, and require a confirmatory observation study that includes factors that influence prognosis such as clinical stage, anti-cancer treatment, comorbidity, and socio-economic characteristics.

## Conclusion

The survival of patients who received home care was significantly better, or at least not worse, than that of patients who received palliative care in a hospital. Although the results of this study are only preliminary, they nevertheless provide important evidence for suggesting home care to patients and their families. A cohort study involving other prognostic factors would confirm this observation to conclude whether palliative home care improves prognosis.

## Abbreviations

ECOG: Eastern cooperative oncology group
QOL: Quality of life
